# Brainstem Nuclei Associated with Mediating Apnea-Induced Respiratory Motor Plasticity

**DOI:** 10.1038/s41598-018-28578-5

**Published:** 2018-08-23

**Authors:** Simon Lui, Zoltan Torontali, Arash Tadjalli, John Peever

**Affiliations:** 1 Centre for Biological Timing and Cognition, University of Toronto, Toronto, Ontario M5S 3G5 Canada; 2 Departments of Cell and Systems Biology, University of Toronto, Toronto, Ontario M5S 3G5 Canada; 3 Department of Physiology, University of Toronto, Toronto, Ontario M5S 3G5 Canada

## Abstract

The respiratory control system is plastic. It has a working memory and is capable of retaining how respiratory stimuli affect breathing by regulating synaptic strength between respiratory neurons. For example, repeated airway obstructions trigger a form of respiratory plasticity that strengthens inspiratory activity of hypoglossal (XII) motoneurons. This form of respiratory plasticity is known as long-term facilitation (LTF) and requires noradrenaline released onto XII motoneurons. However, the brainstem regions responsible for this form of LTF remain unidentified. Here, we used electrophysiology, neuropharmacology and immunohistochemistry in adult rats to identify the brainstem regions involved in mediating LTF. First, we show that repeated airway obstructions induce LTF of XII motoneuron activity and that inactivation of the noradrenergic system prevents LTF. Second, we show that noradrenergic cells in the locus coeruleus (LC), which project to XII motoneurons, are recruited during LTF induction. Third, we show that targeted inactivation of noradrenergic LC cells during LTF induction prevents LTF. And lastly, we show that the nucleus tractus solitarius (NTS), which has known projections to the LC, is critical for LTF because its inactivation prevents LTF. Our results suggest that both the LC and NTS are involved in mediating apnea-induced LTF, and we hypothesize that a NTS → LC → XII circuit mechanism mediates this form of respiratory motor plasticity.

## Introduction

Understanding motoneuron physiology is important because respiratory motoneurons are critical in triggering effective breathing movements. Respiratory motoneurons (e.g., hypoglossal) are sensitive to and modulated by repeated perturbations in central respiratory drive. For example, intermittent episodes of hypoxia or airway obstruction induce a form of respiratory motoneuron plasticity known as long-term facilitation (LTF)^1–4^. LTF results in a long-lasting increase in inspiratory motor outflow to inspiratory muscles (e.g., genioglossus), which may function to facilitate ventilation. We previously demonstrated that repeated airway obstructions trigger LTF of hypoglossal motor outflow (i.e., apnea-induced LTF) and that this form of respiratory plasticity is mediated by a noradrenergic mechanism^4^. Specifically, we found that blocking α1-noradrenergic receptors at the level of hypoglossal motor pool prevented LTF, suggesting that noradrenaline release likely underlies LTF^4^. However, the neural source of noradrenaline responsible for mediating LTF of hypoglossal motoneuron activity remains unidentified. Therefore, we used a combination of electrophysiological, neuro-pharmacological, immunohistochemical and tract-tracing techniques to identify the noradrenergic circuitry that underlies apnea-induced LTF.

We found that pharmacological inactivation of the noradrenergic system prevented LTF, indicating that a noradrenergic mechanism underlies this form of respiratory motor plasticity. Next, we used tract-tracing and c-Fos expression to identify which noradrenergic cells groups are recruited during LTF. We found that noradrenergic cells in the locus coeruleus (LC) are activated during LTF and that they project to hypoglossal motoneurons, suggesting that LC neurons are anatomically and temporally poised to mediate LTF. Importantly, we found that pharmacologically inactivating LC cells during LTF induction blocked its expression. Finally, we found that the nucleus tractus solitarius (NTS), which projects to the LC, is required for mediating apnea-induced LTF because inactivation of the NTS prevents LTF. We hypothesize that a NTS → LC → XII could be the circuit mechanism that mediates this form of respiratory motor plasticity.

## Methods

### Animals

Experiments were performed on anaesthetized, spontaneously breathing young adult male Sprague-Dawley rats. A total of 83 rats, age 8–12 weeks, were used in this study. Rats were purchased from Charles River Laboratories and housed at the University of Toronto, Cell and Systems Biology, Animal Bioscience Facility. Rats were housed in pairs with unlimited access to food and water and maintained on a 12 hour light-dark cycle (lights on at 7 am). Animals were given minimum 1 week to acclimatize to housing conditions before experiments. All experimental procedures in this study were approved by and performed in accordance with both the Canadian Council on Animal Care and University of Toronto Animal Care Committee.

### Surgical procedures

Anaesthesia was introduced with 3.5% isoflurane in a 50/50 oxygen/nitrogen mix, delivered to an induction chamber and maintained via a nose cone at 3% isoflurane. After complete absence of the corneal and foot-withdrawal reflex, a midline incision was made to expose the trachea for a tracheostomy whereby a custom-made T-tube cannula was inserted into the trachea just below the larynx. Anaesthesia was maintained through the T-tube for the remainder of the experiments at 2–2.5% isoflurane set to a flow rate of 1–1.2 L/min. End-tidal CO_2_ was measured in real-time throughout the recording via a custom T-tube cannula connected to an end-tidal CO_2_ analyzer (MicroCapster End-Tidal CO_2_ Analyzer, 15–10000, CWE Inc.). Arterial O_2_ saturation was monitored in real-time using a pulse oximeter designed for rodents, connected to the hind-paw of the animal (MouseOx Pulse Oximeter; STARR Life Sciences Corp.). WINDAQ Waveform Browser software (Dataq Instruments) was used to digitize and analyze O_2_ saturation signals, which were then recorded using Spike2 software. To prevent the accumulation of mucosal secretions in the tracheal T-tube, a subcutaneous injection of atropine sulfate (0.4 mg/kg) was administered. The jugular vein was cannulated for administration of fluids (lactated ringer’s solution at a rate of 1.5 mL/hr) which was administered by a pump driver (BeeHive Pump Controller, MD-1020, BASi). To record upper airway respiratory motor activity, two needle electrodes (F-E2; Grass Technologies) were inserted into the genioglossus muscle, with one electrode on either side of the muscle. To record diaphragm EMG activity, a 1–2 cm midline abdominal incision was made and a custom-made bipolar electrode was fastened onto the fascia of the right diaphragm. All incisions were closed with 9 mm wound clips (Becton Dickinson, 427631, Sparks) to prevent tissue desiccation. In all experiments, rectal temperature was monitored in order to maintain body temperature at 36 ± 0.5 °C via a servo-controlled heating pad (09585; FHC).

### Electrophysiological recordings

To record respiratory activity both genioglossus and diaphragm EMG signals were recorded and amplified between 500–2000 Hz using a Super-Z High Impedance Head Stage and a BMA-400 AC/DC Bioamplifier (10–02010, CWE Inc.). Signals were filtered with a bandpass between 1–3000 Hz for EMG signals sampled at 1000 Hz. End tidal CO_2_ and temperature measurements were sampled at 40 Hz (Spike2 software, 1401 Interface; CED) and digitized (1 kHz; Micro1401; Cambridge Electronic Design). Integrated respiratory EMG activities were quantified using Spike2 software (Cambridge Electronic Design). All signals were stored on a computer for offline analysis.

### Microinjection

In order to manipulate neural activity in the LC and NTS, candidate drugs or vehicle (saline or Ringer’s) were bilaterally injected. To do this, animals were placed in a stereotaxic setup (David Kopf Instruments). Their heads were secured with ear bars and a snout clamp. A 2 cm midline incision was made onto the skin on the dorsal surface of skull. Hydrogen peroxide and saline was used to remove overlying connective tissue and to expose bregma and lamba. A stereotaxic head leveller was used to ensure bregma and lambda were level (David Kopf Instruments). Burr holes were drilled (TX Series, Foredom Electric Co.) bilaterally at the surface of the skull to expose the dura above the LC at coordinates (relative to bregma) 10.0 mm posterior, 1.4 mm lateral, 7.5 mm ventral, or at the NTS at coordinates 13.3 mm posterior, 1.2 mm lateral, 9 mm ventral. Coordinates were guided by the stereotaxic brain atlas by Paxinos and Watson (2004). The dura was then punctured using a 25-gauge sterile hypodermic needle. Stainless steel 28-gauge cannulas (HRS Scientific) connected to a 1 mL gastight syringe (MD-00500, BASi) with FEP Teflon tubing (inner diameter of 0.12 mm; Eicom) were then lowered into the target regions to deliver drug or vehicle solution. The micrinjection was controlled via a syringe pump driver and controller (BeeHive Pump Controllers, MD-1020, BASi) at a rate of 0.1–1 µl/min. Cannula placement was verified by post-mortem histology.

### Drug preparation

All drugs were made immediately before experiments and were dissolved in either Ringer’s or 0.9% saline then filtered (0.2 μm nylon; Thermo Fisher Scientific). Clonidine (clonidine hydrochloride; 266.55 FW; Sigma-Aldrich), an α2 noradrenergic auto-receptor agonist was dissolved in lactated ringers and delivered intravenously (15 µg/kg), or delivered through microinjection (4.8 µg/mL). These concentrations have been shown to be effective at blocking noradrenergic firing or blocking LC activity^5–7^. Lidocaine (2-Diethylamino-N-(2,6-dimethylphenyl) acetamide hydrochloride monohydrate, FW: 288.81 g/mol; Sigma Aldrich), a sodium channel blocker, was prepared as a 5% solution in saline and was used to inactivate cells in the NTS^8^.

### Experimental design

After surgical interventions, rats were left to stabilize for 60-minutes while physiological variables such as respiratory activity (i.e., genioglossus and diaphragm EMG), end-tidal CO_2_, arterial O_2_ saturation and rectal temperature were measured. The 60-minute stabilization period was used to establish baseline conditions for all variables measured. After each intervention, all activities were further recorded for 60–90 minutes. Control experiments of equal duration were also performed to account for potential time-dependant fluctuations in physiological variables.

#### Study 1 – Do repeated obstructive apneas trigger LTF?

To demonstrate that repeated obstructive apneas trigger LTF, rats (n = 14) were exposed to 10 15-second apneas that were separated by 1-minute. Apneas were introduced by occluding the T-tube cannula with hemostats. Obstructions were confirmed by real-time measurements of end-tidal CO_2,_ O_2_ saturation, and magnitude of inspiratory activity of genioglossus and diaphragm EMG activity (for details see Tadjalli *et al*., 2010). Apneas were triggered during end-expiration in order to mimic the obstruction pattern experienced in OSA patients^9,10^. Following repeated apneas, respiratory activity was recorded over 60–90 minutes. Time matched control group of animals without exposure to repeated apneas (n = 11) were used to demonstrate that respiratory activity remained stable throughout the recording period.

#### Study 2 – Does apnea-induced LTF require noradrenaline?

To determine whether a tonic noradrenergic drive is required for apnea-induced LTF, rats (n = 19) were treated with clonidine (15 µg/kg) or vehicle control (lactated ringer’s) through the jugular vein cannulation. Baseline genioglossus and diaphragm EMG activity were recorded for 30 minutes, followed by administration of 10 15-second apneas separated by 1-minute. Respiratory activity was then recorded over the subsequent 60–90 minutes before sacrificing for histological purposes.

#### Study 3 – Does apnea-induced LTF activate noradrenergic neurons?

To determine if noradrenergic structures were activated during apnea-induced LTF, rats (n = 15) were equally divided into 3 groups: rats that exhibited LTF after repeated apneas (i.e. apnea-induced LTF), rats that did not exhibit LTF after repeated apneas (i.e. non-responders), and rats that did not receive apneas (i.e. time-matched controls). All brains were sectioned and stained for c-Fos and tyrosine hydroxylase expression (see, Histology and Cell Quantification below). Cells were counted manually with observers blinded to the experimental treatment.

#### Study 4 – Do LC neurons project directly to the hypoglossal motor pool?

In Study 3, we found that the noradrenergic LC neurons are activated during LTF, therefore we aimed to determine if these neurons project to hypoglossal motoneurons. In 6 rats, we injected a retrograde tracer, cholera toxin B (CtB), into the hypoglossal motor pool. To control for CtB entering cells outside the hypoglossal motor pool, rats (n = 7) had CtB injected 0.2–0.8 mm ventral to the hypoglossal motor pool. Tissue was then stained for tyrosine hydroxylase (TH) to determine whether noradrenergic cells of the LC are co-localized with CtB. Cells were counted manually using fluorescent microscopy.

#### Study 5 – Does bilateral inactivation of the LC prevent apnea-induced LTF?

To determine whether the noradrenergic LC neurons mediate apnea-induced LTF, rats (n = 14) were injected with 200 nL of clonidine (4.8 µg/mL) or vehicle solution into the LC at 0.1 µL/min over 2 minutes, followed by repeated obstructive apneas. Genioglossus and diaphragm EMG activity was recorded for 30-minutes prior to administration with clonidine or vehicle solution to establish a baseline. After administration of clonidine, respiratory activity was recorded for another 30-minutes to establish baseline activity in the presence of clonidine before performing repeated obstructive apneas and recorded for another 60–90 minutes.

#### Study 6 – Does bilateral inactivation of the NTS prevent apnea-induced LTF?

Because apnea-induced LTF is triggered by intermittent modulation of vagal-feedback^4^ and because vagal afferents terminate in the NTS^11^, we aimed to determine if the NTS plays a functional role in mediating apnea-induced LTF. To do this, rats (n = 12) were microinjected with 5% lidocaine dissolved in saline into the NTS, followed by repeated obstructive apneas. Genioglossus and diaphragm EMG activity was recorded for 30 minutes to establish baseline activity. Lidocaine (1 µL) was then injected into the NTS at 1 µL/min. After microinjection, repeated obstructive apneas were delivered in an attempt to elicit apnea-induced LTF. Respiratory activity was recorded for the subsequent 60 minutes.

### Immunohistochemistry

Immunohistochemical staining was performed to verify (1) probe tract locations, (2) cell activity as determined by c-Fos expression, and (3) cell phenotype as determined by tyrosine hydroxylase expression to identify noradrenergic neurons. At the end of each experiment, rats were overdosed with isoflurane (5%) until ventilation ceased, followed by trans-cardiac perfusion with 4% paraformaldehyde (in 0.1 M PB). Brains were extracted and stored in 4% paraformaldehyde overnight, followed by a cryoprotection step by submerging brains into 30% sucrose in 0.1 M PB solution over several days until brains were saturated. Brains were then immersed in Tissue-Tek OCT Compond (Electron Microscope Sciences) and frozen on dry ice. Frozen brains were then sliced in a cryostat (CM3050-S, Leica Microsystems) at 40 μm coronal sections. To determine c-Fos and tyrosine hydroxylase expression, immunohistochemistry was used to identify colocalized expression. Primary antibody rabbit anti-c-Fos (1:5000 dilution, Immunostar, Cat# 26209, lot# 113018B, RRID: AB_572267) was used in conjunction with mouse anti-TH (1:1000 dilution, Immunostar, Cat# 22941, lot# 907001, RRID: AB_572268). After 48 hours of incubation at 4 °C, biotinylated secondary antibodies, biotinylated goat anti-rabbit IgG (1:800 dilution, Vector Laboratories, Cat# BA-1000, lot# Z0619, RRID: AB_2313606) and biotinylated goat anti-mouse IgG (1:600 dilution, Vector Laboratories, Cat# BA-9200, lot# W2206, RRID: AB_2336171) were used. To visualize, an avidin biotin complex (ABC) solution used in conjunction with a 3,3′-diaminobenzidine (DAB) peroxidase kit (DAB Kit, VECTSK4100, Vector Laboratories) to oxidize DAB, providing a brown-black colour in the nuclei of c-Fos positive cells, and NovaRed (NovaRed Kit, VECTSK4800, Vector Laboratories) was used to provide a contrasting red colour to identify noradrenergic cells. Stained tissue was then imaged using Cellsens Slide Scanner (Olympus, FSX100) under bright field at 4x magnification. The location of lesion tracts were plotted on standardized brain maps (Watson and Paxino, 2004).

### Cell quantification

Sections were analyzed under bright-field through a slide scanner (FSX-100 Inverted Microscope, Olympus). To refine our scope, sections were first non-quantitatively analyzed to identify regions with notable changes in c-Fos expression. Noradrenergic regions and areas with unambiguous c-Fos expression were then manually counted with observers blinded to the treatment. A cell was considered c-Fos+ if a cell expressed a black nucleus and excluded cells that expressed nuclei that were light/medium brown (which may or may not be c-Fos+). This level of stringency ensured that we only identified c-Fos+ cells and thereby excluded the possibility of identifying false positive cells. Regions of interest were identified using the rat brain atlas^12^ and counted using ImageJ. Three representative sections were taken across the rostral/caudal axis for each region per animal, and this sampling strategy is based on a recent study that showed that the distribution of LC projections had no specific organization across anterior/posterior or medial/lateral axes^13^.

### Anatomical tracing

In order to determine whether LC neurons directly project to the hypoglossal motor pool, rats were injected with either 200 nL of retrograde tracer cholera toxin B (CtB) conjugated with Alexa Fluor 488 unilaterally into the hypoglossal motor pool (AP 14.5 mm, ML 0.2 mm, DV 9.0 mm) or with 1 µL of viral vector AAV5-hSyn-ChR2(H134R)-mCherry bilaterally into the LC. Rats injected with CtB were sacrificed after 10 days by an overdose of isoflurane and perfused with 4% paraformaldehyde. Rats injected with viral vector were sacrificed 3 weeks later in the same fashion. Brains were extracted, stored in 4% paraformaldehyde overnight, followed by 30% sucrose until brains were saturated. Brains were then frozen and sectioned at 40 µm coronal sections. Sections with CtB were incubated in primary rabbit anti-TH (1:500 dilution, Millipore, Cat# ab152, lot# 2665966, RRID: AB_390204) to identify noradrenergic cells and secondary goat anti-rabbit Cy3 antibodies (1:500, Jackson ImmunoResearch, Cat# 111–167–003, lot#78034, RRID: AB_2313593). They were then counterstained with DAPI (1:1000 dilution, Cell Signalling, Cat# 4083 S, lot# 0007). Sections with viral vector were incubated in primary rabbit anti-mCherry (1:1000, Novus Biologicals, Cat# NBP2-25157, lot# 12016) to enhance reporter protein signals, and secondary goat anti-rabbit Cy3 antibodies (1:500, Jackson ImmunoResearch, Cat# 111-167-003, lot#78034, RRID: AB_2313593). Sections were imaged with the upright fluorescent (AxioImager Z1; Zeiss) and confocal microscope (AxioObserver Z1; Zeiss). Quantification in areas of interests was performed ipsilateral to the injection site.

### Data analysis

Peak integrated inspiratory genioglossus and diaphragm EMG amplitudes as well as respiratory frequency were quantified on a breath-by-breath basis in 60-sec intervals during all experiments. Inspiratory amplitude and respiratory frequency were expressed as a percent change from baseline ± standard error of the mean (SEM). Baseline values for inspiratory amplitude and respiratory frequency were acquired during the 60-sec period before each experimental intervention. Data were quantified and expressed before (i.e., baseline) and at 15, 30, 45 and 60-min after experimental interventions. Equivalent time points were quantified and expressed in experiments serving as controls without the apnea-induced intervention. Animals were determined to exhibit LTF if they met two specific criteria: (1) genioglossus inspiratory amplitude was two standard deviations above baseline levels 60-min after recurrent apneas; and, (2) summated genioglossus inspiratory amplitude averaged over 60-min was two standard deviations above baseline levels. If an animal failed either criterion they were considered not to exhibit LTF and were therefore excluded from the “LTF group”. Arterial O_2_ saturation and end-tidal CO_2_ values were also expressed as a percentage change from baseline ± SEM and presented at 15, 30, 45 and 60-min after each experimental intervention or at equivalent time points in control experiments. Each presented data point was an average over 60-sec.

### Statistical analysis

The specific statistical tests used for each experiment are stated within the results section. All datasets passed normality. Comparisons for each respiratory variable within a treatment across time (e.g. repeated apneas on genioglossus amplitude at baseline, 15, 30, 45, and 60 minute time points) were made using a one-way repeated measure analysis of variance (one-way RM ANOVA) and *post hoc* comparisons were performed using the Dunnett test. Comparisons between treatments for each respiratory variable were made using a two-way RM ANOVA with *post hoc* Bonferroni test to infer statistical significance. Cell counts between groups were compared using a one-way ANOVA and *post hoc* comparisons were performed using the Dunnett test. A two-tailed unpaired t-test was used to determine significance in mean expiration duration of the Hering-Breuer reflex following NTS inactivation. All statistical analyses used GraphPad Prism (Prism v5.0, GraphPad). Data are presented as a mean ± standard error of the mean.

### Data availability

All data generated or analysed during this study are included in this published article.

### Significance statement

The respiratory control system is plastic. It has a working memory and is capable of retaining how respiratory stimuli affect breathing by regulating synaptic strength between respiratory neurons. Repeated airway obstructions – similar to those experienced in obstructive sleep apnea – trigger a form of respiratory memory that strengthens inspiratory activity of hypoglossal motoneurons. This form of respiratory memory is known as long-term facilitation (LTF) and is mediated by noradrenergic-dependent mechanism. But, the neural mechanism responsible for this form of LTF remains unidentified. Here, we identify two potential brainstem regions that are associated with mediating apnea-induced LTF. We found that recurrent obstructive apneas modulate vagal feedback, which activates cells in the nucleus of the solitary tract. These cells in turn can manipulate locus coeruleus cells, which episodically release noradrenaline onto hypoglossal motoneurons thereby triggering hypoglossal motoneuron plasticity and hence LTF.

## Results

### Repeated obstructive apneas trigger LTF

**Figure 1:**
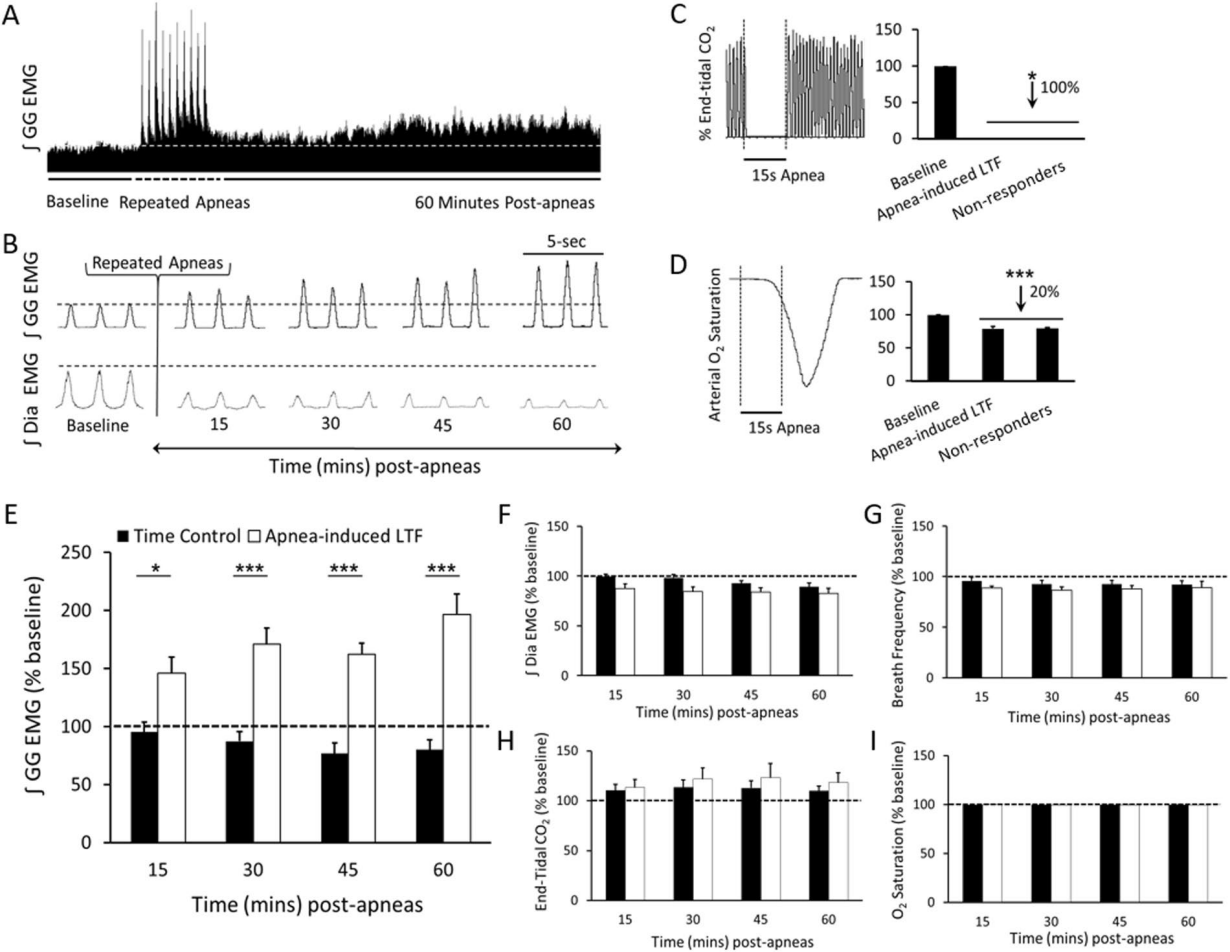
Repeated obstructive apneas elicit LTF of the genioglossus motor activity. (**A**) Integrated inspiratory genioglossus (GG) motor output recorded from an anaesthetized spontaneously breathing rat, depicting baseline genioglossus amplitude and the subsequent increase in EMG amplitude following repeated apneas (i.e., LTF). (**B**) High-temporal resolution EMG traces showing genioglossus (top) and diaphragm (bottom) activity at baseline, 15, 30, 45, and 60 min after repeated apneas. (**C**) Group data showing inspiratory genioglossus activity time matched control (i.e. no apneas; black bars) and intermittent apnea groups (white bars) at 15, 30, 45, and 60 min. Dotted line represents baseline activity. Intermittent apneas induced an increase in inspiratory genioglossus amplitude peaking at 96 ± 17% (2-way RM ANOVA, *F* = 11.22, p < 0.0001). A raw trace showing end-tidal CO_2_ (**D**) and arterial O_2_ saturation (**E**) before, during, and after an apnea (left), and group data (right) showing undetectable end-tidal CO_2_ levels during apnea indicating complete occlusion, and arterial O_2_ saturation levels reduced by 20 ± 4% following an apnea (n = 3, one-way RM ANOVA, p < 0.0001). Although intermittent apneas triggered a robust increase in inspiratory genioglossus activity (i.e., LTF), this same intervention had no significant effect on either (**F**) inspiratory diaphragm (Dia) amplitude (2-way RM ANOVA, *F* = 2.433, p = 0.0557) or (**G**) breath frequency (2-way RM ANOVA, *F* = 2.113, p = 0.0880). (**H**) Both end-tidal CO_2_ (2-way RM ANOVA, *F* = 0.4221, p = 0.7921) and (**I**) O_2_ saturation levels remained constant across the 60 min recording period (2-way RM ANOVA, *F* = 1.043, p = 0.4160). Data is presented as means ± SEM. *Denotes a significant difference (p < 0.05) from baseline.

Our first objective was to demonstrate that repeated obstructive apneas trigger LTF of genioglossus motor output. We found that recurrent airway occlusions (10, 15-s apneas each separated by 1-min) triggered a robust and sustained increase in inspiratory genioglossus muscle activity that peaked at 96 ± 17% above baseline levels by 60 min (2-way RM ANOVA, *F* = 11.22, p < 0.0001; Fig. 1A,B,C). Apneas, which were confirmed by a total loss of expired CO_2_ and a drop in O_2_ saturation (Fig. 1D,E), only amplified the magnitude of inspiratory genioglossus activity; they had no long-term effect on respiratory frequency or diaphragm activity (2-way RM ANOVA, *F* = 2.113, p = 0.0880 and *F* = 2.433, p = 0.0557, breath frequency and inspiratory diaphragm amplitude, respectively; Fig. 1F,G). LTF of inspiratory genioglossus amplitude was not attributable to changes in end-tidal CO_2_ or changes to O_2_ saturation as both variables remained consistent between groups over the 60-min recording period. (2-way RM ANOVA, *F* = 0.4221, p = 0.7921 and *F* = 1.043, p = 0.4160, for end-tidal-CO_2_ and O_2_ saturation, respectively; Fig. 1H,I).

To demonstrate that respiratory activity remained stable throughout the recording period, genioglossus and diaphragm activity were recorded in a control group that experienced no recurrent apneas. We found that genioglossus amplitude, breath frequency, end-tidal CO_2_ and O_2_ saturation remained stabled during the 60 min time-window (RM ANOVA, genioglossus: *F* = 1.33, p = 0.2757; breath frequency: *F* = 1.988, p = 0.1148; ET-CO_2_: *F* = 1.485, p = 0.2272; O_2_ saturation: *F* = 1.546, p = 0.2776; Fig. 1**)**, indicating that genioglossus activity does not change over the 60 min recording period and that LTF of genioglossus muscle activity is attributable to recurrent apneas *per se*. A decrease in diaphragm amplitude over time was observed (RM ANOVA, *F* = 4.996, p = 0.0026), but was in line with animals given recurrent apneas (RM ANOVA, *F* = 6.408, p = 0.0007).

### Disruption to noradrenergic drive prevents apnea-induced LTF

Having shown that recurrent apneas trigger LTF of inspiratory genioglossus activity, our next goal was to identify the neurotransmitter mechanisms underlying this form of respiratory motor plasticity. Previously, we showed that apnea-induced LTF requires α1-receptor activation within the hypoglossal motor pool^4^, suggesting that a noradrenergic mechanism mediates LTF. To demonstrate that LTF of genioglossus activity is indeed noradrenergic-dependent, we inactivated noradrenergic cell activity by systemically delivering clonidine, which is an α2 autoreceptor agonist that functions to reduce or eliminate noradrenergic cell discharge^5,7^.

**Figure 2:**
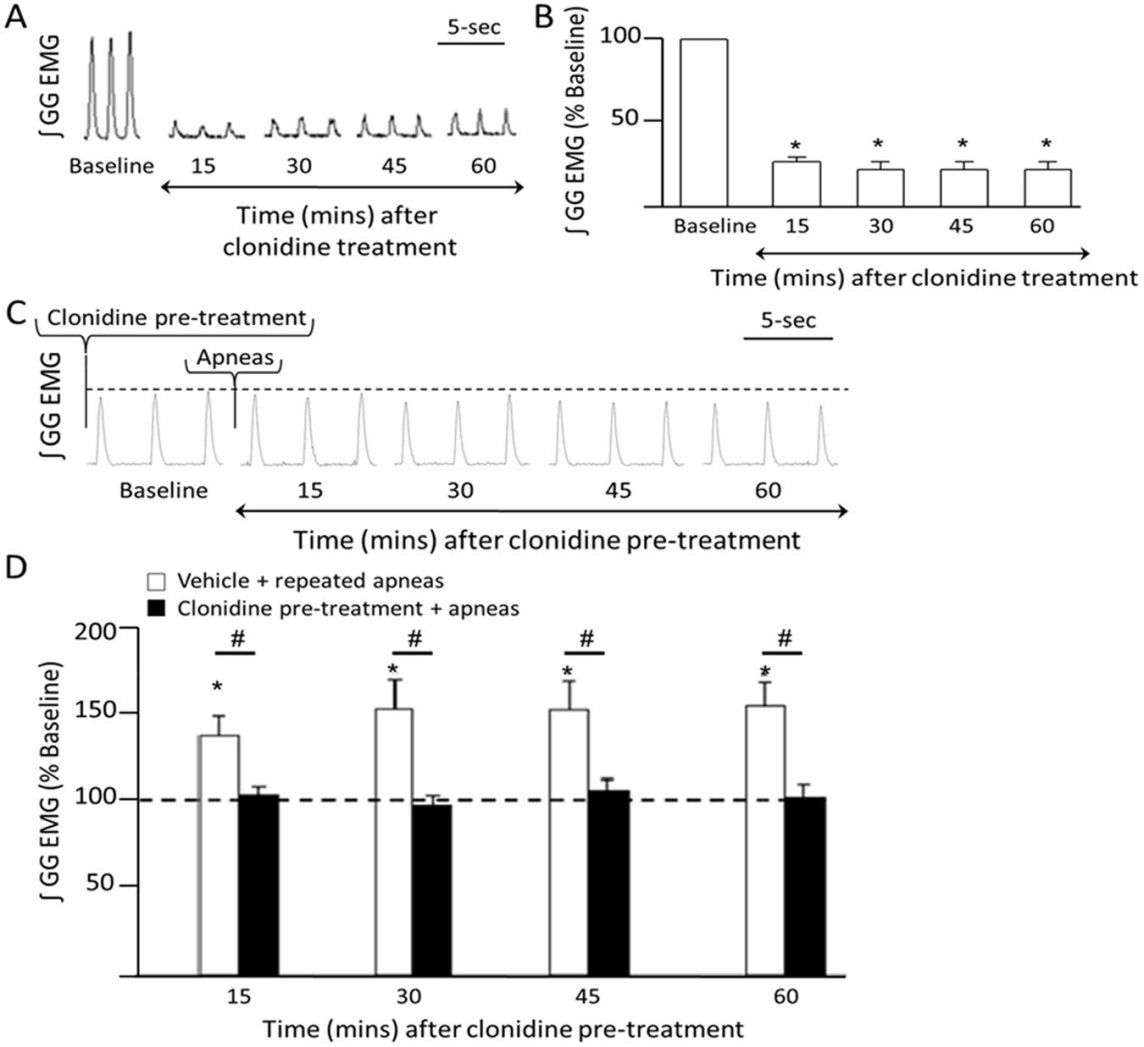
α2 auto-receptor mediated processes affect basal genioglossus muscle tone and expression of LTF. (**A**) A representative integrated inspiratory genioglossus (GG) EMG activity before (i.e., baseline) and 15, 30, 45, and 60 min after systemic clonidine (15 ug/kg i.v.) recorded from an anaesthetized spontaneously breathing rat. (**B**) Group data (n = 6) demonstrating that clonidine administration reduced inspiratory genioglossus activity to 11 ± 5% of its baseline activity (RM ANOVA, p < 0.05), presumably by reducing noradrenergic drive onto hypoglossal motoneurons. Group data are expressed as percentage change from baseline ± SEM. (**C**) A representative EMG trace of integrated inspiratory genioglossus activity during baseline and 15, 30, 45, and 60 min following intermittent apneas after clonidine pre-treatment. (**D**) Group data demonstrating that in vehicle treated rats, repeated apneas elicited LTF of inspiratory genioglossus activity (white bars) (RM ANOVA, p < 0.05), however in the presence of clonidine, plasticity is absent (black bars) (RM ANOVA, p > 0.05). Group data are expressed as percentage change from baseline ± SEM. Dotted line represents baseline activity. *Denotes a significant difference (p < 0.05) from baseline. ^#^Denotes a significant difference (p < 0.05) between the indicated data sets.

First, we showed that clonidine (15 ug/kg i.v) application reduced baseline activity of inspiratory genioglossus output to 11 ± 5% of its baseline activity (RM ANOVA, p < 0.05; Fig. 2A,B), suggesting that clonidine not only suppressed noradrenergic system activity, but that an endogenous noradrenergic drive onto hypoglossal motoneurons facilitates inspiratory genioglossus activity. In contrast, clonidine application did not affect diaphragm activity (RM ANOVA, p > 0.05; data not shown), indicating that a noradrenergic drive does not contribute to diaphragm activity. We also observed a 24 ± 2% reduction in breath frequency during the 60-min recording period following clonidine administration (RM ANOVA, p < 0.05; data not shown), suggesting that noradrenergic neurons stimulate breathing frequency as has been previously noted^14^. Next, we showed that systemic clonidine injection completely blocked apnea-induced LTF of inspiratory genioglossus activity when compared to baseline genioglossus activity post clonidine pre-treatment (RM ANOVA, p > 0.05). Compared to the vehicle control group (i.e., Ringer’s), which triggered a 55 ± 6.1% increase above baseline in inspiratory genioglossus activity following recurrent airway occlusions, animals treated with clonidine show no such increase in genioglossus activity following intermittent apneas (RM ANOVA, vehicle vs clonidine: p < 0.05; Fig. 2C,D). These findings suggest that noradrenergic activity is required for promoting apnea-induced LTF of inspiratory genioglossus activity.

### Activation of noradrenergic LC neurons correlates with LTF

**Figure 3:**
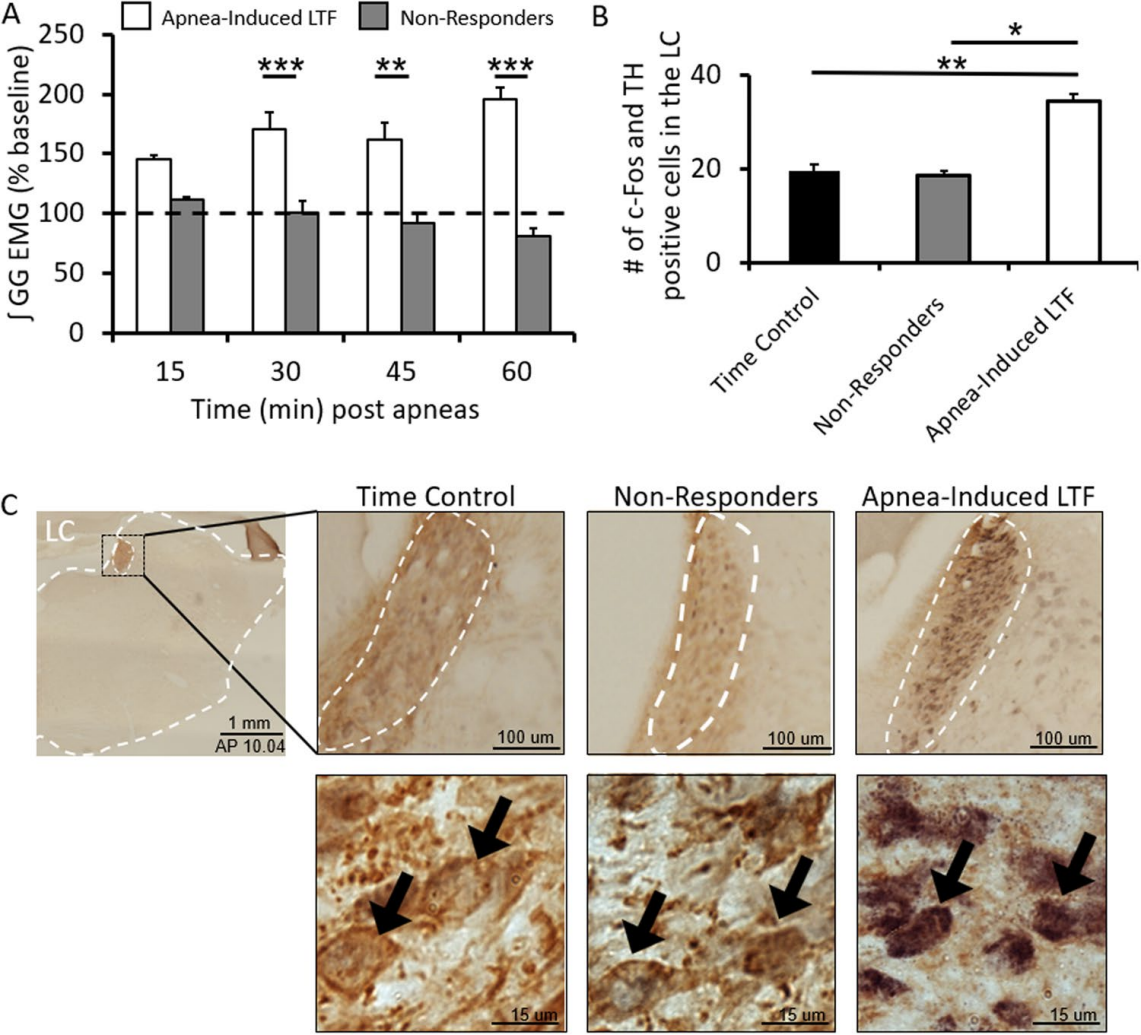
LC activation correlates with apnea-induced LTF. (**A**) Group data showing inspiratory genioglossus amplitude after recurrent apneas in responders (i.e. apnea-induced LTF; white bars) and non-responders (i.e. apnea without LTF; grey bars) at 15, 30, 45, and 60 min. Dotted line represents baseline activity. Intermittent apneas produced LTF with inspiratory genioglossus amplitude peaking at 96 ± 17% but non-responders did not exhibit LTF following the repeated apnea intervention (2-way RM ANOVA, *F* = 6.686, p = 0.0002). (**B**) Group data showing LC activity in the no apnea (time control, n = 5, black bar), apneas without LTF (“non-responders”, n = 5), and apnea-induced LTF (n = 5) groups. Animals that exhibited LTF had LC activity increased by 176 ± 7% (unpaired t-test, time control vs apnea-induced LTF: *t*_(51)_ = 3.334, p = 0.0016). This increase was not attributed to the recurrent apneas because an identical intervention but absent LTF did not display an increase in double-labelled cells. In fact, animals exhibiting LTF had LC activity increase by 186 ± 7% compared to animals given an identical protocol but did not exhibit LTF (unpaired t-test, apnea-induced LTF vs non-responders: *t*_(56)_ = 2.103, p = 0.0399). No difference was observed between the time control and non-responder group. (**C**) An example of LC activity represented by c-Fos expression in the time control, non-responders, and apnea-induced LTF groups. c-Fos expression was identified by cell nuclei stained black with DAB and tyrosine hydroxylase (TH) expression was identified by red-brown NOVA-Red stain to phenotype noradrenergic cells. Magnified examples of LC neurons (black arrows) that are c-Fos negative, TH-positive (time control and non-responders) and LC neurons that are double-positive (last panel). Data is presented as means ± SEM. *Denotes a significant difference (p < 0.05).

Having shown that LTF requires a noradrenergic mechanism, our next aim was to identify the potential source of noradrenaline mediating apnea-induced LTF. We did this by quantifying c-Fos expression within noradrenergic cell groups (i.e., A1, A2, A5, A6 [LC], and A7) following induction of LTF and compared expression levels with a control group (i.e., animals not exposed to recurrent airway occlusions). Compared to the time control group, apnea-induced LTF increased c-Fos expression in the LC by 176 ± 7% (unpaired t-test, *t*_(51)_ = 3.334, p = 0.0016; Fig. 3B,C**)**; however, we found no evidence for changes in c-Fos expression in the other noradrenergic cells groups (i.e., A1, A2, A5 and A7; Table 1), suggesting that apnea-induced LTF only activates noradrenergic cells in the LC.

**Table 1:** The LC is the only noradrenergic cell group to exhibit c-Fos expression following recurrent apneas. (−) = 0–2 cells, (+) = 3–15 cells, (++) 16–26 cells, (+++) = 26 + cells.

Nuclei	Time Control	Non-Responders	Apnea-Induced LTF	*p*-values
NTS	+	++	+	*p* = 0.3702
Hypoglossal	−	−	−	NA
A1	++	+	++	*p* = 0.1430
A2	+	+	+	*p* = 0.1393
A5	+	+	+	*p* = 0.1935
**A6 (LC)**	**++**	**++**	**+++**	***p*** = **0.0044**
A7	−	−	−	NA

### Activation of noradrenergic cells in the LC is LTF-dependent

**Figure 4:**
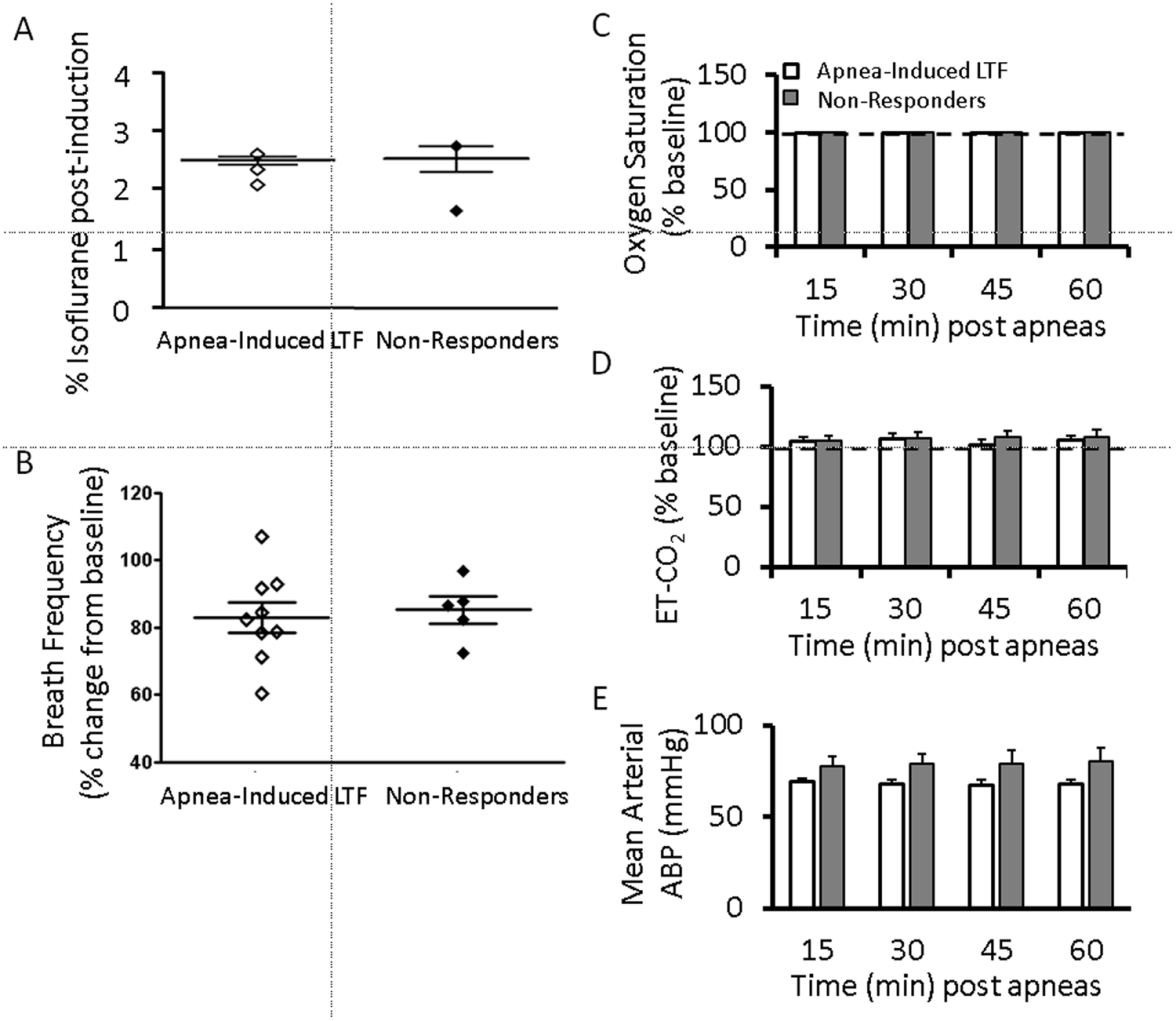
Levels of anesthesia, expired CO_2_, O_2_ saturation and blood pressure do not correlate with LTF. A comparison of the percentage of isoflurane used to maintain anesthesia in animals that exhibited LTF after repeated apneas (i.e., “apnea-induced LTF”; white diamonds) and animals that did not exhibit LTF after apneas (i.e., “non-responders”; black diamonds) (**A**). Breath frequency of each animal expressed as a percent change from baseline did not differ between animals exhibiting apnea-induced LTF and non-responders (unpaired t-test, *t*_(12)_ = 0.3227, p = 0.7525). Comparisons of O_2_ saturation levels (**C**), end-tidal CO_2_ levels (**D**) and mean arterial blood pressure (BP) (**E**) between animals exhibiting apnea-induced LTF and non-responders. The lack of difference in levels of anaesthesia, ET-CO_2_ (2-way RM ANOVA, *F* = 0.7049, p = 0.5933), O_2_ saturation (2-way RM ANOVA, *F* = 0.5439, p = 0.7054) and blood pressure in the apnea-induced LTF and non-responder groups (**A**–**E**) suggest these factors are unlikely contributors to the lack of LTF in the non-responder group. Data is presented as means ± SEM.

While the preceding experiments suggest that LTF activates LC cells, LC activity is also increased by hypoxic, hypercapnia and airway occlusion^15–18^, which makes it difficult to link the observed increases in LC activity with LTF induction. Therefore, we wanted to determine if increased LC activity is attributable to LTF *per se*, so we examined LC c-Fos expression levels in which recurrent apneas triggered LTF and compared them with cases in which recurrent apneas did not trigger LTF (i.e. non-responders) (Fig. 3A). We were able to make such comparisons because LTF does not always occur following repeated apneas. Specifically, we found that recurrent apneas triggered LTF 65% of the time (i.e., 35% of the time repeated apneas did not induce LTF and were categorized as non-responders), and this rate of plasticity induction is in line with classic long-term potentiation (LTP) studies which report that LTP only occurs 50 to 90% of the time (i.e. LTP does not occur 10 to 50% of the time)^19,20^. Nonetheless, we first wanted to ensure that the lack of LTF expression in the non-responder group was not influenced by changes in anaesthesia depth. Therefore, in addition to corneal and toe pinch reflexes used throughout the experiment, we quantified the percentage of inhaled isoflurane at the beginning and end of each experiment and recorded respiratory frequency as an index of anaesthesia depth in the LTF and No LTF groups. In both cases, animals were induced at 3.5% isoflurane and reduced after tracheostomy to maintain anaesthesia. Maintenance varied between animals but averaged around 2.5% isoflurane (Fig. 4A). Similarly, no difference in breath frequency at 60-min post apneas and presumably anaesthesia depth was observed (unpaired t-test, *t* = 0.3227, p = 0.7525) (Fig. 4B). Next, we compared end-tidal CO_2_ and oxygen saturation levels between groups to ensure blood gases were not confounding factors in LTF expression. Neither ET-CO_2_ nor oxygen saturation levels were significantly different between groups (2-way RM ANOVA, LTF responders vs non-responders, ET-CO_2_: *F* = 0.7049, p = 0.5933. O_2_ saturation: *F* = 0.5439, p = 0.7054) **(**Fig. 4C,D**)**. We also compared mean arterial blood pressure between groups and found no observable difference between animals that developed LTF and those that did not (Fig. 4E). Limited numbers of non-responders with blood pressure recordings prevented a statistical comparison.

We then compared c-Fos expression between animals that exhibited LTF (apnea-induced LTF) and those that did not (non-responders). Compared to non-responders, we found that apnea-induced LTF increased c-Fos expression in the LC by 186 ± 7% (apnea-induced LTF vs non-responders: unpaired t-test, *t*_(56)_ = 2.103, p = 0.0399; Fig. 3B,C). This observation suggests that activation of noradrenergic cells in the LC is LTF-dependent and is not associated with the hypoxia and hypercapnia associated with apneic stimuli.

### Noradrenergic LC neurons project to hypoglossal motoneurons

**Figure 5:**
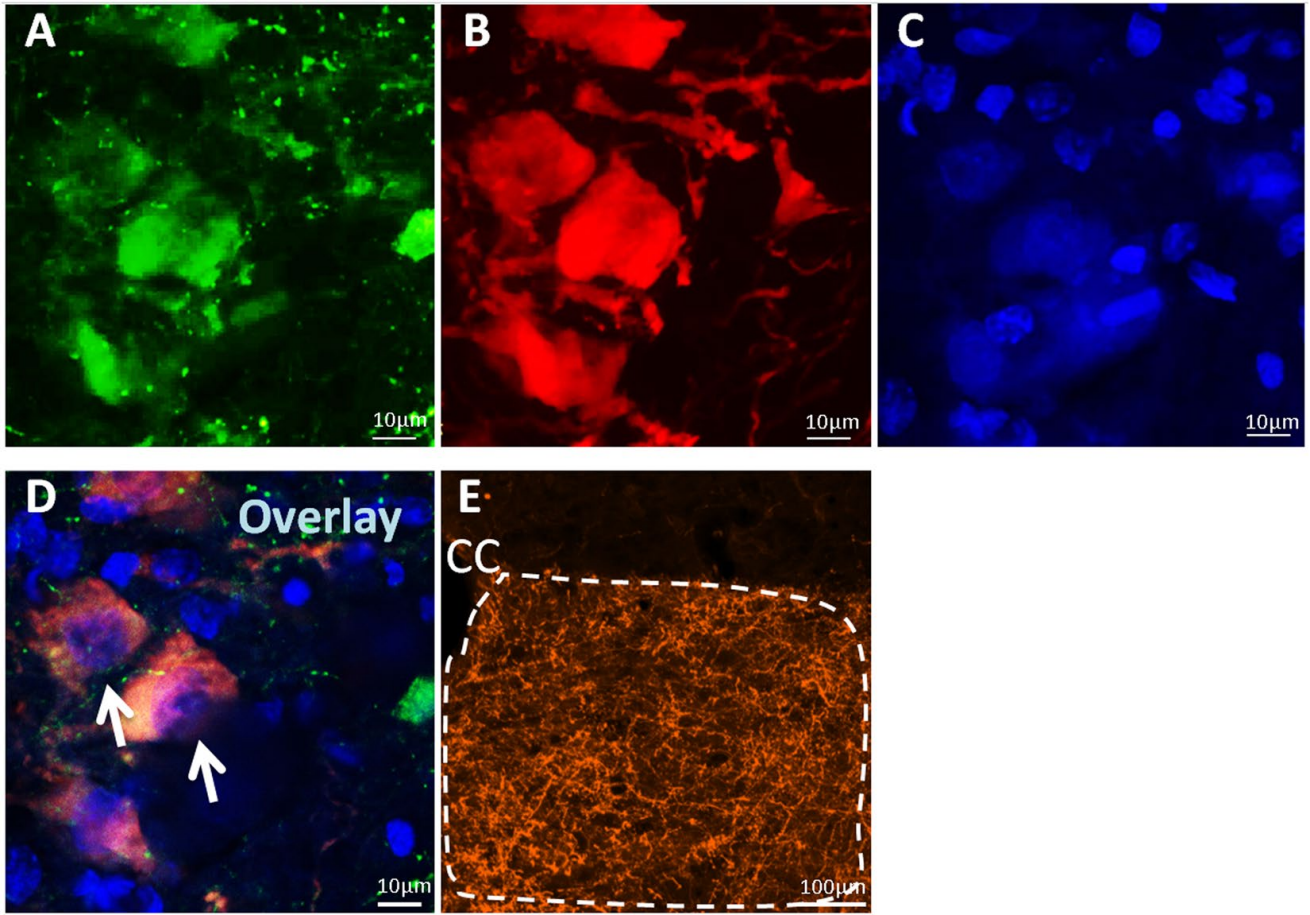
Noradrenergic LC neurons have direct projections to the hypoglossal motor pool. (**A**) An example of an LC neuron expressing cholera toxin B (CtB) conjugated with AlexaFluor-488 (green) 10 days after CtB was injected into the hypoglossal motor pool (n = 6). Cells were counterstained for tyrosine hydroxylase (**B**) and DAPI (**C**) to identify noradrenergic cells. Triple-labelled neurons (**D**) are indicated by the white arrows, suggesting noradrenergic cells in the LC directly project to the hypoglossal motor pool. To verify this connection, AAV5-hsyn-ChR2(H134R)-mCherry injected into the LC showed visible axon terminals at the level of the hypoglossal motor pool (**E**). CC, central canal.

Having shown that noradrenergic LC cell activity is associated with apnea-induced LTF, we next wanted to verify that these cells project to and synapse on neurons within the hypoglossal motor pool. To do this we injected cholera toxin B (CtB) conjugated with AlexaFluor-488 into the hypoglossal motor pool in order to determine if noradrenergic cells (i.e., TH+ noradrenergic cells) project to the hypoglossal motor pool. We found that CtB injection within the hypoglossal motor pool labeled 71 ± 9% of noradrenergic cells within the LC (n = 6; Fig. 5A–D). However, we found that injections that were 0.2–0.8 mm ventral to the hypoglossal motor pool did not result in CtB labeling of noradrenergic LC cells (n = 7; data not shown), demonstrating that noradrenergic LC cell labeling is selective to CtB injections in the hypoglossal motor pool. To further demonstrate that LC cells project to hypoglossal motoneurons, we also injected an adeno-associated virus (AAV) to trace LC axonal projections to the hypoglossal motor pool. Specifically, when AAV5-hSyn-ChR2(H134R)-mCherry was injected into the LC, we found clear evidence of mCherry labeled axon terminals within the hypoglossal motor pool (Fig. 5E). We also observed axonal projections to other documented respiratory regions including the nucleus ambiguous (Amb)^21^, the facial motor nucleus (VII)^21–23^, and the parabrachial nucleus (PBN)^23^ (data not shown). Together, these results indicate that noradrenergic LC cells project to and synapse within the hypoglossal motor pool, presumably on hypoglossal motoneurons.

### Bilateral inactivation of the LC prevents apnea-induced LTF

**Figure 6:**
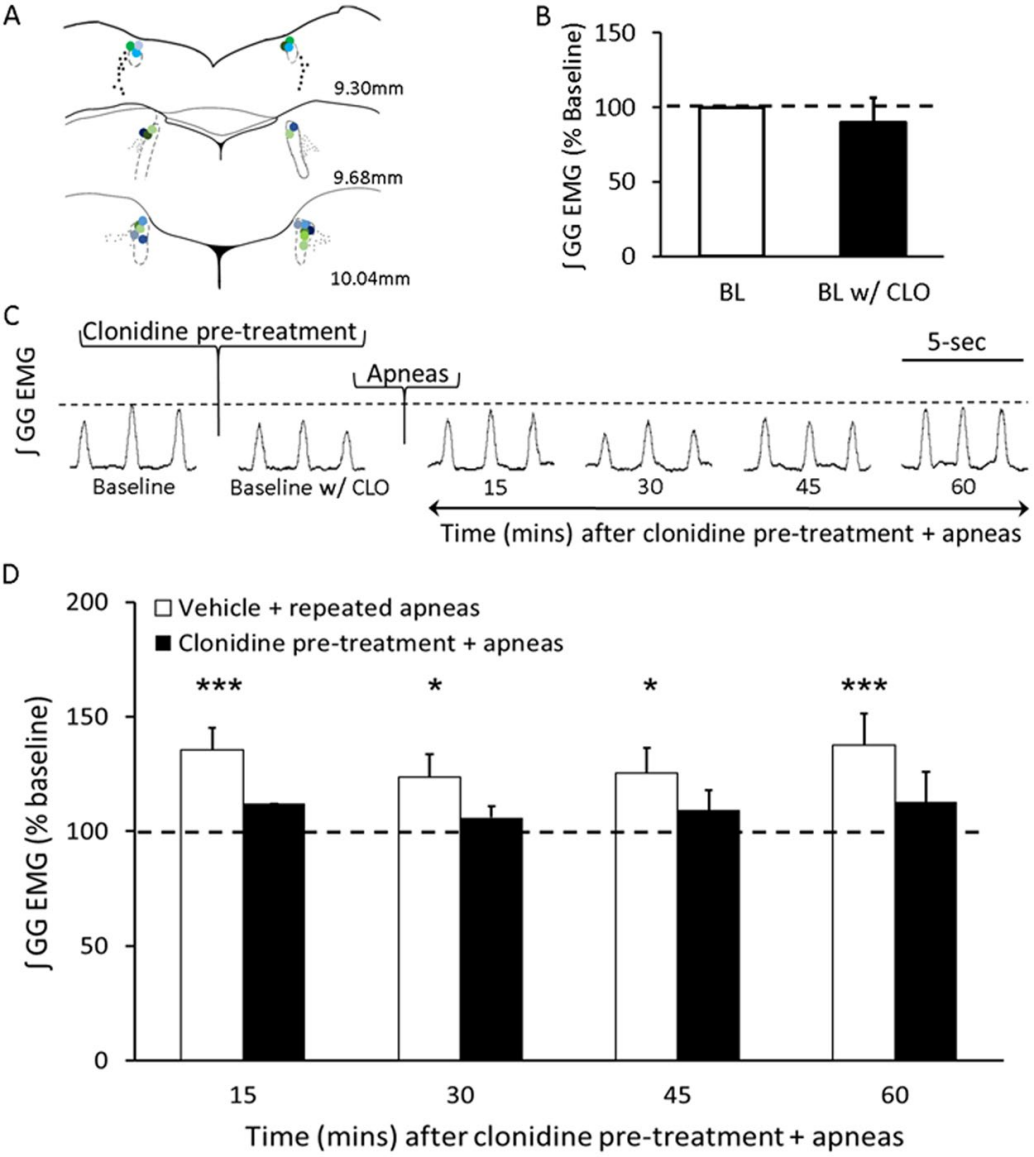
Inactivation of the LC prevents apnea-induced LTF. (**A**) Probe tract locations in the LC following bilateral microinjection of clonidine. Green circles represent clonidine-treated animals and blue circles represent vehicle controls. (**B**) Group data showing inspiratory genioglossus (GG) activity before (BL) is not significantly different after clonidine application at the LC (BL w/CLO) (unpaired t-test, n = 6, *t*_(10)_ = 0.5490, p = 0.5950). (**C**) A representative EMG trace of integrated inspiratory genioglossus (GG) activity before LC inactivation (baseline), after LC inactivation (baseline w/CLO), and 15, 30, 45, and 60 min after repeated apneas. (**D**) Group data showing inspiratory genioglossus activity in the vehicle-treated (white bars) and clonidine-treated (black bars) animals at 15, 30, 45, and 60 min following repeated apneas. Dotted line represents average baseline activity. Intermittent apneas increased inspiratory genioglossus amplitude by 40 ± 13% (i.e., LTF) (RM ANOVA, *F* = 6.476, p = 0.0008) in vehicle-treated animals, but in the presence of clonidine apnea-induced LTF was absent (RM ANOVA, *F* = 0.5975, p = 0.6686). Data are presented as means ± SEM. *Denotes a significant difference (p < 0.05).

Having demonstrated that a noradrenergic mechanism mediates LTF and the noradrenergic LC cells are activated during LTF and that they project to the hypoglossal motor pool, our next step was to determine if noradrenergic LC cells mediate LTF of genioglossus muscle activity. To do this, we pharmacologically inactivated LC cells by focally injecting clonidine into the left and right LC nuclei. However, before this we wanted to verify that bolus fluid injections alone did not influence the expression of apnea-induced LTF, therefore, we injected an equal volume of vehicle (Ringer’s) into the left and right LC nuclei 30 min before recurrent airway occlusions. We found that vehicle injections had no effect on expression of apnea-induced LTF (RM ANOVA, *F* = 6.476, p = 0.0008) with inspiratory genioglossus activity increasing up to 40 ± 3% above baseline levels. However, compared to vehicle injections, we found that inactivating noradrenergic LC cells by bilateral clonidine injection completely prevented apnea-induced LTF (RM ANOVA, *F* = 0.5975, p = 0.6686; Fig. 6C,D), with inspiratory genioglossus activity remaining with baseline levels during the 60 min period following injection. This observation suggests that noradrenergic LC cells are required for driving the expression of apnea-induced LTF. Post-mortem histology was used to confirm that injections were located within the left and right LC nuclei (Fig. 6A).

To ensure that clonidine applied to the LC did not suppress baseline genioglossus activity we compared baseline genioglossus activity before and after clonidine application, and found no significant effect of clonidine on genioglossus activity (unpaired t-test, *t*_(10)_ = 0.5490, p = 0.5950; Fig. 6B). This suggests that the absence of LTF after clonidine treatment does not stem from suppressed genioglossus activity.

### The NTS is required for apnea-induced LTF

**Figure 7:**
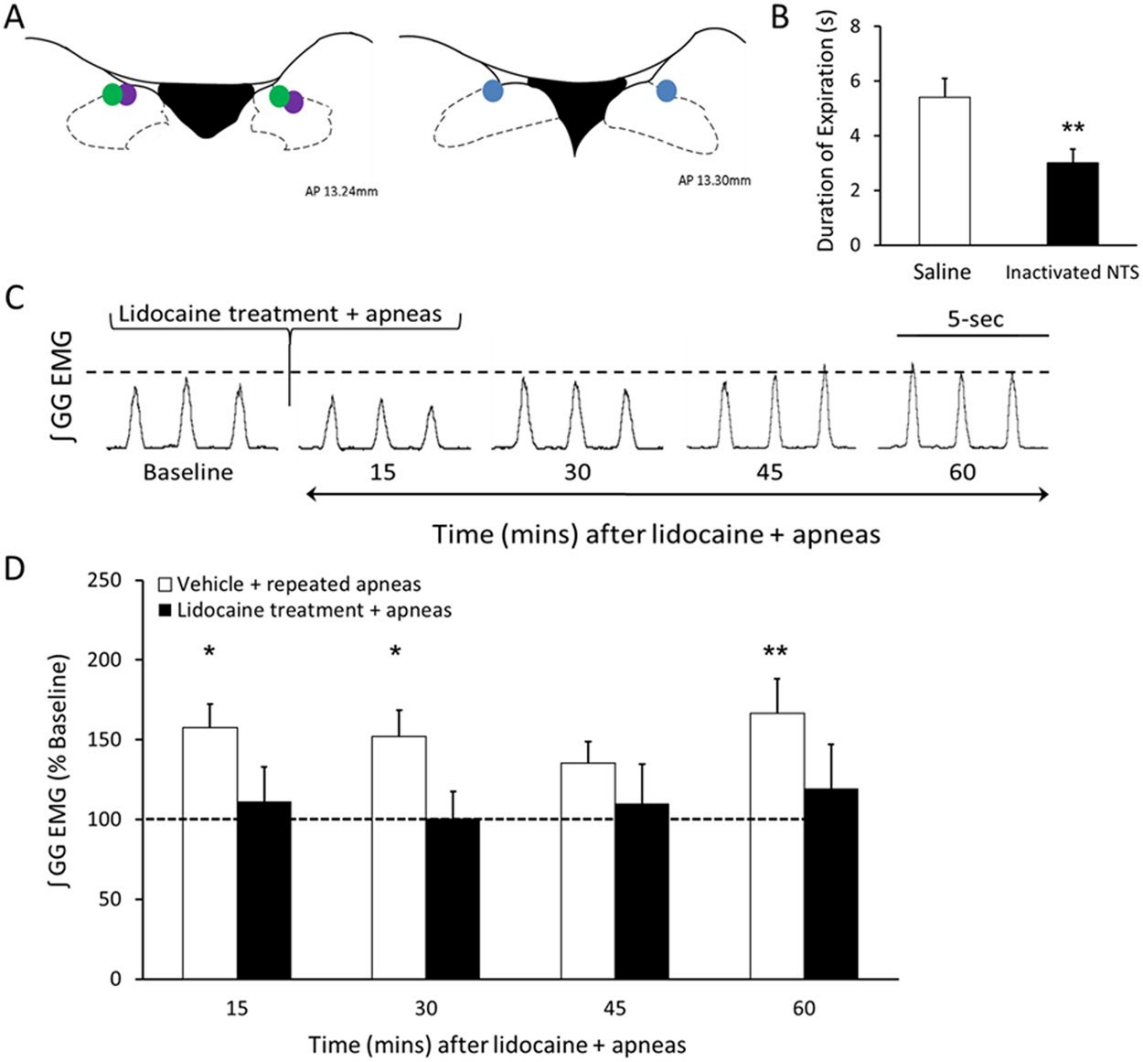
Inactivation of the NTS prevented apnea-induced LTF. (**A**) Probe tract locations in the NTS following bilateral microinjection of lidocaine. (**B**) Mean expiratory duration (seconds) of the Hering-Breuer reflex before (white bar) and after (black bar) inactivation of the NTS (unpaired t-test, n = 7, t_12_ = 3.227, p = 0.0073). (**C**) A representative EMG trace of integrated inspiratory genioglossus (GG) activity before (i.e., baseline) and after NTS inactivation at 15, 30, 45, and 60 min following repeated apneas. (**D**) Group data showing inspiratory genioglossus activity in the vehicle-treated (white bars) and lidocaine-treated (black bars) animals at 15, 30, 45, and 60 min following repeated apneas. Dotted line represents average baseline activity. Intermittent apneas induced a peak of 67 ± 21% increase in inspiratory genioglossus amplitude (i.e., LTF) (RM ANOVA, *F* = 4.310, p < 0.0091) in vehicle-treated animals, but in the presence of lidocaine, NTS cells were inactivated effectively abolishing apnea-induced LTF (RM ANOVA, *F* = 0.3331, p = 0.8517). Data are presented as means ± SEM. *Denotes a significant difference (p < 0.05).

Having shown that noradrenergic LC cell activity is required for LTF expression, we next wanted to identify a mechanism by which recurrent apneas recruit and activate LC cells. Because vagal feedback is required for apnea-induced LTF^4^ and because vagal afferents terminate in the NTS^11^ and project to and modulate LC activity^24,25^, we hypothesized that the NTS is required for triggering apnea-induced LTF. Therefore, we pharmacologically inactivated cells in the NTS by bilaterally injecting lidocaine into the left and right NTS before repeatedly occluding the airway. However, we first wanted to verify 1) that drug perfusion could successfully inactivate the NTS, and 2) that the bolus injection itself did not disturb LTF. To demonstrate NTS inactivation was effective, we occluded the airway after inspiration to induce the Hering-Breuer reflex. We found that NTS inactivation significantly reduced the mean expiratory duration of the Hering-Breuer reflex (unpaired t-test, n = 7, t_12_ = 3.227, p = 0.0073; Fig. 7B). Next, we injected saline into the left or right NTS 1 min before repeatedly occluding the airway to demonstrate that LTF was not disturbed by the bolus injection itself. We found that saline injection had no effect on expression of apnea-induced LTF (RM ANOVA, *F* = 4.310, p < 0.0091), with inspiratory genioglossus activity increasing up to 67 ± 21% above baseline levels. However, compared to saline injection, we found that inactivating NTS cells by bilateral lidocaine injection completely blocked apnea-induced LTF (RM ANOVA, *F* = 0.3331, p = 0.8517; Fig. 7C,D), with inspiratory genioglossus activity remaining within baseline levels during the 60 min recording period. This finding suggests that the NTS is required for apnea-induced LTF. Post-mortem histology was used to confirm that injections were located within the left and right NTS (Fig. 7A).

Because the NTS is involved in respiratory control^26,27^ we wanted to ensure that NTS inactivation did not disrupt eupneic breathing, so we analyzed diaphragm activity and breath frequency after NTS inactivation. Neither inspiratory diaphragm amplitude nor breath frequency were significantly altered over 60 mins after NTS inactivation and recurrent apneas (*F* = 0.9301, p = 0.4712, *F* = 1.158, p = 0.3659, diaphragm activity and breath frequency, respectively, RM ANOVA; data not shown).

## Discussion

This study is scientifically important because it contributes to our understanding of circuit and transmitter mechanisms underlying respiratory motor plasticity. Here, we identified two brainstem regions that underlie apnea-induced LTF of genioglossus motor activity. We demonstrate that noradrenergic cells in the LC are activated following LTF induction and that pharmacological inactivation of LC activity prevents LTF, suggesting that noradrenaline release - likely from the LC - is required for LTF expression. We also show that inactivation of NTS activity prevents apnea-induced LTF, suggesting that these cells are critical for apnea-induced LTF expression.

### A noradrenergic mechanism underlies apnea-induced LTF

An endogenous noradrenergic drive plays a defined role in facilitating motor output from hypoglossal motoneurons during wakefulness, sleep and during anaesthesia^28^. Noradrenergic drive also plays a role in promoting LTF since we and others recently found that apnea-induced LTF of genioglossus motor output requires noradrenaline release onto hypoglossal motoneurons^3^. Here, we confirm that noradrenergic activity is required for promoting apnea-induced LTF of genioglossus activity by showing that both pharmacological inactivation of general noradrenergic system activity and targeted inactivation of LC cells prevented LTF expression. Given that blockade of α1-adrenergic receptors within the hypoglossal motor pool prevented LTF, it is likely that repeated obstructive apneas trigger noradrenaline release from one (or more) of the brainstem noradrenergic cell groups (likely the LC) that project to hypoglossal motoneurons.

### Noradrenergic cells in the LC are active during LTF

Hypoglossal motoneurons receive noradrenergic inputs from several different cells groups, including the A5, A7 and LC (Fig. 5), indicating that LTF could be triggered by noradrenaline onto hypoglossal motoneurons from one or more of these noradrenergic nuclei. Using c-Fos expression as an index of noradrenergic activity, we found that noradrenergic cells in the LC are activated by apnea-induced LTF, suggesting that they could be the neural substrate that triggers noradrenaline release onto hypoglossal motoneurons.

However, unlike a previous report^3^, we found no evidence to indicate that noradrenergic cells in either the A5 or A7 nuclei are activated following LTF induction. Methodological differences are the likely reason for the differences between our observations. Here, we probed c-Fos expression levels 90 min after LTF induction, whereas, Song and Poon examined expression levels 20 min after LTF induction. We quantified c-Fos expression 90 min following LTF induction because c-Fos levels are maximal 60–90 min following stimulus-induced neuronal activation^29,30^. Differences between our results and those of Song and Poon could also stem from the use of different anaesthetics, which are known to influence c-Fos expression^31,32^.

Our results indicate that noradrenergic LC cell activity is associated with LTF expression, but because LC cells are responsive to obstructive apnea, hypercapnia and hypoxia^15,16,18^, it is possible that changes in LC activity following recurrent apneas could result for apneic and/or hypercapnia/hypoxic stimuli rather than LTF itself. To indirectly test this possibility, we examined c-Fos in cases where recurrent apneas did not elicit LTF of inspiratory genioglossus activity (i.e., 5 of 14 cases). We found no evidence for increased c-Fos expression in LC cells when recurrent apneas did not trigger LTF, which contrasts with the robust increase in c-Fos expression when recurrent apneas triggered LTF (Fig. 3B,C). This observation suggests that activation of noradrenergic LC cells is not caused by recurrent apneas or chemical stimuli associated with them; instead, LC activation is linked to LTF induction *per se*. We interpret these findings to indicate that increased LC activity is the causal mechanism that triggers LTF of genioglossus activity because: (1) noradrenergic cells are only active when LTF is triggered by apneas (i.e., increased c-Fos in animals that exhibit LTF; Fig. 3B,C); (2) that apnea-induced LTF is noradrenaline-dependent (Fig. 2D); (3) that noradrenergic LC neurons project to XII motoneurons (Fig. 5); and, (4) inactivation of LC cells during apneas prevents LTF (Fig. 6D). Based on these pieces of evidence and the fact that apneas act through vagal afferents and NTS neurons, which in turn project to LC neurons^25,33,34^, we claim that recurrent apneas activate LC neurons to trigger apnea-induced LTF. However, these studies do not identify why LC neurons are activated in the LTF group, but not in the “No LTF” group; this would be the subject of future studies. But, as presented and interpreted, our data are consistent with previously studies showing that increases in c-Fos expression can be used to identify cells associated with inducing other forms of plasticity, such as granule cells and their role in mediating long-term potentiation (LTP) in the cerebellum^35^, or parvalbumin basket cells and their role in regulating plasticity^36^.

### Noradrenergic cells in the LC are required for LTF expression

Our current and previous results^3,4^ suggest that apnea-induced LTF is mediated by a noradrenergic-dependent mechanism. However, the neural source of noradrenaline responsible for mediating LTF remained, until now, unidentified. Here, we provide evidence indicating that LC cells are the likely source of noradrenaline release onto hypoglossal motoneurons that ultimately underlies LTF of inspiratory genioglossus activity.

First, we provide correlative evidence showing that LC cells are recruited during LTF (Fig. 3B,C). We also show that noradrenergic LC cells project to and innervate hypoglossal motoneurons (Fig. 5), confirming previous reports that LC neurons innervate hypoglossal motoneurons^34^. Taken together, these observations suggest that recurrent obstructive apneas activate LC cells, which in turn release noradrenaline onto hypoglossal motor neurons thereby triggering LTF. However most importantly, we show that targeted pharmacological inactivation of LC cells prevents the expression of apnea-induced LTF. And this latter observation provides support for the causal link between apnea-induced LC activation and its contribution to triggering of LTF of inspiratory genioglossus activity. Although noradrenergic LC cells have previously been shown to facilitate motoneuron activity and hence muscle tone^37,38^, our results are physiologically important because they suggest that noradrenaline release from LC cells is also involved in facilitating motor outflow by inducing LTF of motoneuron activity.

Although focal inactivation of LC activity blocked LTF expression, it did not reduce baseline inspiratory genioglossus activity (Fig. 6B), suggesting that LC cells release negligible amounts of noradrenaline onto hypoglossal motoneurons during anaesthetized conditions. Because previous studies show that inspiratory hypoglossal motor outflow is facilitated by an endogenous noradrenergic drive during both natural behaviors and anaesthesia, it is likely that other noradrenergic nuclei (e.g., A5 and/or A7 cell groups) provide this drive^3,39^. One caveat to our study is that LC inhibition has been shown to increase blood pressure by ~10 mmHg^40^, potentially influencing the absence of LTF observed. However, LTF can still be generated even when blood pressure is elevated by 10–15 mmHg^41^, suggesting that increased blood pressure does not prevent apnea-induced LTF expression. We suggest that the absence of LTF following either systemic or focal (i.e., in the LC) clonidine treatment is not due to blood pressure changes but is due to reduced noradrenergic drive to hypoglossal motoneurons.

### The NTS contributes to LTF expression

We previously showed that vagal feedback is critical for triggering apnea-induced LTF^4^. Because vagal afferents terminate in the NTS^11^ and project to and modulate LC activity^24,25^, we hypothesized that the NTS is required for apnea-induced LTF. We found that pharmacological inactivation of NTS cells prevented expression of LTF of inspiratory genioglossus motor outflow, indicating that vagal feedback induced by recurrent obstructive apneas likely stimulates the NTS, and that the NTS is critical for LTF induction.

Although our NTS inactivation studies indicate that the NTS is critical for apnea-induced LTF, we found no evidence for elevated c-Fos expression levels in the NTS during LTF. Even though c-Fos expression is a useful method for identifying neural circuits associated with behavior^42^, it has limitations^15,29,43^. For example, some neurons (e.g., respiratory motoneurons) do not express c-Fos when activated by respiratory stimuli (e.g., hypercapnia)^15^. Therefore, it is likely that recurrent apneas caused NTS activation, but the relatively mild nature of this stimulus was insufficient to induce detectable changes in c-Fos expression levels. However, our loss-of-function data clearly suggest that NTS activity is critical for triggering apnea-induced LTF.

### Brainstem regions associated with apnea-induced LTF

**Figure 8:**
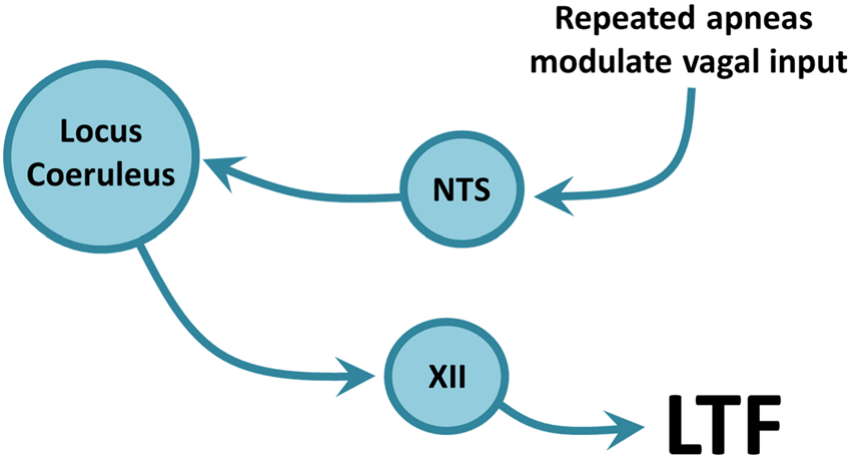
Hypothesized circuitry responsible for LTF. Repeated obstructive apneas modulate vagal afferent activity, which terminates in the nucleus tractus solitarius (NTS). We hypothesize that cells in the NTS send excitatory projections to the noradrenergic cells of the locus coeruleus (LC), which in turn extend axons directly to the hypoglossal (XII) motor pool to modulate hypoglossal (and therefore genioglossus) activity, effectively triggering LTF. We hypothesize that this is the neural circuit underlying LTF of inspiratory genioglossus motor output.

To date, most studies have examined the molecular pathways that trigger respiratory LTF within motoneurons^3,44–47^, but our current study is important because it identifies an intact circuit underlying apnea-induced respiratory motor plasticity. Based on our results we propose that recurrent obstructive apneas – similar to those experienced in obstructive sleep apnea (OSA) – modulate vagal feedback, which activates NTS cells. Although the interaction between NTS and LC cells was not directly tested, we hypothesize that NTS cells in turn manipulate LC cells, which episodically releases noradrenaline onto hypoglossal motoneurons thereby triggering motoneuron plasticity and hence LTF of inspiratory genioglossus motor activity (Fig. 8).

Understanding the circuit mechanisms underlying LTF of hypoglossal motoneuron activity is relevant to human health because reduced genioglossus muscle tone during sleep is a contributing factor in OSA^48^. Although respiratory LTF has been identified in humans, its potential role in mitigating reduced genioglossus muscle tone in OSA remains to be determined. A recent study demonstrated LTF in humans could not be elicited using a chemical trigger (hypercapnic hypoxia)^49^. Our results are particularly important in this context because we show that LTF depends on noradrenergic LC cell activity, independent of hypercapnia/hypoxia. Furthermore, LC cells are largely inactive during sleep^50–52^, suggesting that the circuits required for LTF are dysfunctional during sleep. Therefore, developing strategies to activate noradrenergic activity during sleep may be a viable tool for engaging LTF and hence mitigating OSA^3^.
